# Challenges in the Measurement of Staffing: A Review of Reviews

**DOI:** 10.1093/geroni/igab046.555

**Published:** 2021-12-17

**Authors:** Franziska Zúñiga, Magdalena Osinska, Catherine Blatter, Michael Simon

**Affiliations:** University of Basel, Basel, Basel-Stadt, Switzerland

## Abstract

The question concerning the relationship of staffing and quality of care of residents in residential long-term care (LTC) has been explored extensively; however, no consistent evidence has been brought forth so far. Inconsistent measurement of staffing might hinder this research field to move forward. We assessed its measurement in a narrative review of reviews that explore the staff – quality of care relationship. We identified 12 systematic reviews, covering 1960 to May 2018. Most studies included had a cross-sectional design, were performed in the USA and worked with secondary, administrative data (e.g., OSCAR). Comparability of studies was limited by diverse definitions and measurement methods for staffing, including data about grade-mix, number of staff, and staff-resident ratios. We suggest performing international multi-case studies to compare and contrast LTC staffing and develop appropriate international common data elements. Logic models support the description of the expected relationship between staffing aspects and quality.

